# Supplementary material for: Fertility return after hormonal contraceptive discontinuation and associated factors among women attended Family Guidance Association of Ethiopia Dessie model clinic, Northeast Ethiopia: A cross-sectional study
Source: PLoS One. 2023 Jul 11;18(7):e0287440. doi: 10.1371/journal.pone.0287440 (PMC10335688; doi:10.1371/journal.pone.0287440)
Supplement: Attachment — Submitted filename: PONE-D-21-25440-Reviewed.pdf [file pone.0287440.s003.pdf]

PLOS ONE

Fertility return after contraceptive discontinuation and associated factors among women attended Family Guidance Association of Dessie model clinic, Northeast Ethiopia: a cross-sectional study

--Manuscript Draft--

**Manuscript Number:**	PONE-D-21-25440
**Article Type:**	Research Article
**Full Title:**	Fertility return after contraceptive discontinuation and associated factors among women attended Family Guidance Association of Dessie model clinic, Northeast Ethiopia: a cross-sectional study
**Short Title:**	Fertility return after contraceptive discontinuation among women
**Corresponding Author:**	Yitayish Damtie, MPH<br>Wollo University<br>Dessie, Ethiopia ETHIOPIA
**Keywords:**	Fertility, delayed fertility return, infertility, women, Ethiopia
**Abstract:**	Background: Women who use hormonal contraception faces delayed or impaired fertility upon discontinuation. There was limited evidence on fertility return after contraceptive discontinuation in the study area. Hence this study assessed fertility return after contraceptive discontinuation and associated factors among pregnant women attending Family Guidance Association (FGA) Dessie model clinic, Northeast Ethiopia, 2018.<br>Methods: A cross-sectional study was conducted on 423 samples selected by using systematic random sampling. Data were collected by face to face interview using a pretested and structured questionnaire and reviewing client records. Data were entered using Epi Data version 3.1 and analyzed using SPSS version 23. Both bi-variable and multivariable binary logistic regressions were used to identify predictors of delayed fertility return. Adjusted odds ratio (AOR) along with a 95 % Confidence Interval (CI) was used to measure the strength and the direction of the association and statistical significance was declared at a P-value less than 0.05.<br>Result: The proportion of delayed fertility return among currently pregnant women after discontinuation of any contraceptive methods was 11.4% (95% CI; (8-14.4%)). The proportion of delayed fertility return among Depo-Provera, implant, Uterine Contraceptive Device (IUCD), and Oral Contraceptive Pill (OCP) users was (25%), (0.9%), 0%, (2.2%) respectively. Age, (AOR= 1.15, (95% CI; (1.01, 1.30)) and using Depo-Provera (AOR=6.22, (95% CI; 5.23, 13.3)) had a significant association with delayed fertility return.<br>Conclusions: The proportion of delayed fertility returns among women after the discontinuation of any contraceptive methods was consistent with other studies. But the proportion of delayed fertility return among implant, IUCD, and OCP users was low. Age and using Depo-Provera had a positive association with delayed fertility return. Health care providers should provide detailed family planning counseling for their clients.<br>Keywords: Fertility, delayed fertility return, infertility, women, Ethiopia
**Order of Authors:**	Yitayish Damtie, MPH
	Bereket Kefale
	Mastewal Arefaynie
	Melaku Yalew
	Bezawit Adane
**Additional Information:**	
**Question**	**Response**
**Financial Disclosure**	The author(s) received no specific funding for this work.

Powered by Editorial Manager® and ProduXion Manager® from Aries Systems Corporation

Enter a financial disclosure statement that describes the sources of funding for the work included in this submission. Review the submission guidelines for detailed requirements. View published research articles from *PLOS ONE* for specific examples.<br><br>This statement is required for submission and **will appear in the published article** if the submission is accepted. Please make sure it is accurate.<br><br>**Unfunded studies**<br>Enter: *The author(s) received no specific funding for this work.*<br><br>**Funded studies**<br>Enter a statement with the following details:<br>• Initials of the authors who received each award<br>• Grant numbers awarded to each author<br>• The full name of each funder<br>• URL of each funder website<br>• Did the sponsors or funders play any role in the study design, data collection and analysis, decision to publish, or preparation of the manuscript?<br>• **NO** - Include this sentence at the end of your statement: *The funders had no role in study design, data collection and analysis, decision to publish, or preparation of the manuscript.*<br>• **YES** - Specify the role(s) played.<br><br>* typeset	
**Competing Interests**<br><br>Use the instructions below to enter a competing interest statement for this submission. On behalf of all authors, disclose any competing interests that could be perceived to bias this work—acknowledging all financial support and any other relevant financial or non-financial competing interests.<br><br>This statement is required for submission and **will appear in the published article** if the submission is accepted. Please make	The authors have declared that no competing interests exist.

sure it is accurate and that any funding sources listed in your Funding Information later in the submission form are also declared in your Financial Disclosure statement.<br><br>View published research articles from *PLOS ONE* for specific examples.<br><br>**NO authors have competing interests**<br><br>Enter: *The authors have declared that no competing interests exist.*<br><br>**Authors with competing interests**<br><br>Enter competing interest details beginning with this statement:<br><br>*I have read the journal's policy and the authors of this manuscript have the following competing interests: [insert competing interests here]*<br><br>* typeset	
**Ethics Statement**<br><br>Enter an ethics statement for this submission. This statement is required if the study involved:<br><br>• Human participants<br>• Human specimens or tissue<br>• Vertebrate animals or cephalopods<br>• Vertebrate embryos or tissues<br>• Field research<br><br>Write "N/A" if the submission does not require an ethics statement.<br><br>General guidance is provided below. Consult the submission guidelines for detailed instructions. **Make sure that all information entered here is included in the Methods section of the manuscript.**	Ethical Clearance was taken from the Ethical Review Committee (ERC) of Wollo University College of Medicine and Health Sciences. An official letter was written from the school of Public Health to FGA Dessie Model Clinic head to get permission. After explaining the purpose of the study, verbal informed consent was taken from each participant before the data collection. They were informed that participating in the study was voluntary and the right to withdraw from the study at any time during the interview was assured. Privacy and confidentiality of information they gave was secured at all levels.

**Format for specific study types**<br><br>**Human Subject Research (involving human participants and/or tissue)**<br>• Give the name of the institutional review board or ethics committee that approved the study<br>• Include the approval number and/or a statement indicating approval of this research<br>• Indicate the form of consent obtained (written/oral) or the reason that consent was not obtained (e.g. the data were analyzed anonymously)<br><br>**Animal Research (involving vertebrate animals, embryos or tissues)**<br>• Provide the name of the Institutional Animal Care and Use Committee (IACUC) or other relevant ethics board that reviewed the study protocol, and indicate whether they approved this research or granted a formal waiver of ethical approval<br>• Include an approval number if one was obtained<br>• If the study involved *non-human primates*, add *additional details* about animal welfare and steps taken to ameliorate suffering<br>• If anesthesia, euthanasia, or any kind of animal sacrifice is part of the study, include briefly which substances and/or methods were applied<br><br>**Field Research**<br><br>Include the following details if this study involves the collection of plant, animal, or other materials from a natural setting:<br>• Field permit number<br>• Name of the institution or relevant body that granted permission	
**Data Availability**<br><br>Authors are required to make all data underlying the findings described fully available, without restriction, and from the time of publication. PLOS allows rare exceptions to address legal and ethical concerns. See the PLOS Data Policy and FAQ for detailed information.	Yes - all data are fully available without restriction

A Data Availability Statement describing where the data can be found is required at submission. Your answers to this question constitute the Data Availability Statement and **will be published in the article**, if accepted.<br><br>**Important:** Stating 'data available on request from the author' is not sufficient. If your data are only available upon request, select 'No' for the first question and explain your exceptional situation in the text box.<br><br>Do the authors confirm that all data underlying the findings described in their manuscript are fully available without restriction?	
**Describe where the data may be found in full sentences. If you are copying our sample text, replace any instances of XXX with the appropriate details.**<br><br>• If the data are **held or will be held in a public repository**, include URLs, accession numbers or DOIs. If this information will only be available after acceptance, indicate this by ticking the box below. For example: *All XXX files are available from the XXX database (accession number(s) XXX, XXX.*).<br>• If the data are all contained **within the manuscript and/or Supporting Information files**, enter the following: *All relevant data are within the manuscript and its Supporting Information files.*<br>• If neither of these applies but you are able to provide **details of access elsewhere**, with or without limitations, please do so. For example:<br><br>*Data cannot be shared publicly because of [XXX]. Data are available from the XXX Institutional Data Access / Ethics Committee (contact via XXX) for researchers who meet the criteria for access to confidential data.*<br><br>*The data underlying the results presented in the study are available from (include the name of the third party*	The datasets used for the current study are available from the corresponding author on reasonable request.

and contact information or URL).<br>• This text is appropriate if the data are owned by a third party and authors do not have permission to share the data.<br><br>* typeset	
Additional data availability information:	

Fertility return after contraceptive discontinuation and associated factors among women attended Family Guidance Association of Dessie model clinic, Northeast Ethiopia: a cross-sectional study

Yitayish Damtie[1*], Bereket Kefale[1], Mastewal Arefaynie[1], Melaku Yalew[1], Bezawit Adane[2]

[1]Department of Reproductive and Family Health, School of Public Health, College of Medicine and Health Sciences, Wollo University, Ethiopia

[2] Department of Epidemiology and Biostatistics, School of Public Health, College of Medicine and Health Sciences, Wollo University, Ethiopia

* Correspondence: Yitayish Damtie

Tel: +251943517982

Email Address: yitutile@gmail.com

Abstract

Background: Women who use hormonal contraception faces delayed or impaired fertility upon discontinuation. There was limited evidence on fertility return after contraceptive discontinuation in the study area. Hence this study assessed fertility return after contraceptive discontinuation and associated factors among pregnant women attending Family Guidance Association (FGA) Dessie model clinic, Northeast Ethiopia, 2018.

Methods: A cross-sectional study was conducted on 423 samples selected by using systematic random sampling. Data were collected by face to face interview using a pretested and structured questionnaire and reviewing client records. Data were entered using Epi Data version 3.1 and analyzed using SPSS version 23. Both bi-variable and multivariable binary logistic regressions were used to identify predictors of delayed fertility return. Adjusted odds ratio (AOR) along with a 95 % Confidence Interval (CI) was used to measure the strength and the direction of the association and statistical significance was declared at a P-value less than 0.05.

Result: The proportion of delayed fertility return among currently pregnant women after discontinuation of any contraceptive methods was 11.4% (95% CI; (8-14.4%)). The proportion of delayed fertility return among Depo-Provera, implant, Uterine Contraceptive Device (IUCD), and Oral Contraceptive Pill (OCP) users was (25%), (0.9%), 0%, (2.2%) respectively. Age, (AOR= 1.15, (95% CI; (1.01, 1.30)) and using Depo-Provera (AOR=6.22, (95% CI; 5.23, 13.3)) had a significant association with delayed fertility return.

Conclusions: The proportion of delayed fertility returns among women after the discontinuation of any contraceptive methods was consistent with other studies. But the proportion of delayed fertility return among implant, IUCD, and OCP users was low. Age and using Depo-Provera had

a positive association with delayed fertility return. Health care providers should provide detailed family planning counseling for their clients.

Keywords: Fertility, delayed fertility return, infertility, women, Ethiopia

Introduction

Globally, around 15% of couples experience infertility [1]. It can be defined as the inability of couples to conceive within 12 months of unprotected sexual intercourse [2]. Even though many effective, safe and reversible modern contraceptives were discovered in the world, women who use hormonal contraception face delayed fertility return upon discontinuation [3-5]. Delayed fertility return or infertility among women is attributable to their previous contraceptive use. For example, different evidences showed that tubal infertility was higher among previous IUCD users [6, 7]. Another study also showed that Post-pill amenorrhea was observed among previous OCP users [8-12].

Delayed fertility return or impaired fertility had a significant impact on women's health. It lower contraceptive use irrespective of actual desire, it leads to divorce, stigma and discrimination, isolation, intimate partner violence, murder, mental health disorders, and suicide [3-5, 13-15].

Studies showed different factors have been associated with delayed fertility return. These include socio-demographic factors (age) [16], behavioral factors (smoking, alcohol drinking [17], obstetric factors (parity, gravidity) [16, 18] and contraceptive related factors (duration of contraceptive use, type of contraceptive) [17, 19, 20].

Ethiopian government adopts Sustainable Development Goal (SDG) and Growth and Transformation Plan (GTP II) to decrease MMR below 199 and 267/100,000 live births respectively [21, 22]. Addressing the family planning need in Ethiopia is expected to avert 12,800 maternal and 1.1 million child deaths [23]. Even though universal access to family planning can

reduce maternal deaths by 40% and infant mortality by 10% [24, 25], according to Ethiopian Demographic and Health Survey (EDHS) 2016, only 36% of currently married women used contraceptives and 22% of women have an unmet need for family planning [26]. One of the reasons for not using contraceptive methods might be due to fertility delay associated with contraceptive utilization.

There was limited evidence regarding fertility return after contraceptive discontinuation in the study area. So, this study aimed to assess fertility return after contraceptive discontinuation and associated factors among pregnant women attending FGA Dessie Model Clinic.

Material and methods

Study area, study design, and participants

An institution-based cross-sectional study was conducted in FGA Dessie Model Clinic from May 1- 30/2019. The source population was all pregnant women attending the Antenatal Care (ANC) unit of FGA Dessie Model Clinic whereas; the study population was all pregnant women attending the ANC unit of FGA Dessie Model Clinic during the study period. All pregnant women coming for ANC during the data collection period were included and those women who became pregnant without using any contraceptive method and due to contraceptive failure were excluded from the study.

Sample size and sampling procedure

The sample size was determined by using single population proportion formula by considering the proportion of delayed fertility return as 50% since no study was done in Ethiopia, 95% confidence level and 5% margin of error. Thus, the final sample size after adding a 10 % non-response rate

became 423**.** Finally, samples were selected by using a systematic random sampling technique until reaching the final sample size.

Data collection procedures and measurements

Data were collected by face to face interview using a pretested and structured Amharic version questionnaire taken from p[illegible]ious similar works of literature. The questionnaire was composed of Socio-demographic factors, behavioral factors, obstetric, contraceptive, disease, and nutritional related factors. Two trained nurses have collected the data with the supportive supervision of one supervisor and principal investigators. Data collectors and supervisor were trained for two days on the objective of the study, the content of the questionnaire and the data collection procedure. Before the data collection, the questionnaire was pretested on 22 study participants at Dessie health center and based on feedback obtained from the pretest, a necessary modification was done. During the study period, the collected data were checked continuously on a daily basis for completeness by the supervisor and principal investigators.

The outcome variable was the fertility return. A Woman said to experience delayed fertility return if she became pregnant after 12 months following discontinuation of any modern contraceptive methods (Depo-Provera, OCP, IUCD, and implant) and didn't experience delayed fertility return if she became pregnant within 12 months following discontinuation of any modern contraceptive methods [19, 27].

Statistical analysis

Data were coded and entered into Epi Data version 3.1 and exported to SPSS version 23 for analysis. Descriptive statistics such as frequency, percentage and median with Interquartile Range

(IQR) were carried out. Bi-variable binary logistic regression was performed and variables with a p-value of less than 0.25 were transported to multivariable logistic regression. Multicollinearity was checked using standard error and Hosmer and Lemeshow goodness of test was used to check model fitness. Variables with a P-value less than 0.05 and AOR with a 95% confidence interval non-inclusive of one were considered as statistically significant predictors of delayed fertility return in the final model.

Ethical approval

Ethical Clearance was taken from the Ethical Review Committee (ERC) of Wollo University College of Medicine and Health Sciences. An official letter was written from the school of Public Health to FGA Dessie Model Clinic head to get permission. After explaining the purpose of the study, verbal informed consent was taken from each participant before the data collection. They were informed that participating in the study was voluntary and the right to withdraw from the study at any time during the interview was assured. Privacy and confidentiality of information they gave was secured at all levels.

This manuscript was organized and written according to strengthening the Reporting of Observational Studies in Epidemiology (STROBE) 2007 (v4) Statement checklist for cross-sectional studies (S1 table).

Results

Socio-demographic characteristics

In this study, a total of 402 pregnant women were involved and making a response rate of 94.5%. The median age of the respondents was 28 years with IQR of 4 years. Three hundred eighty (94.5%) of pregnant women were married, 22(5.5%) of pregnant women were single and divorced

and 361(89.8%) of pregnant women were Amhara in their ethnicity respectively. Two hundred sixteen (53.7%) pregnant women were orthodox tewahido followers, 203(50.5%) of women earn less than or equal to 5,000 Ethiopian birr and 392 (97.5%) of women live in urban areas respectively. One hundred ninety-six (48.8%) of pregnant women were educated up to college and university level and 143(35.6%) of women were government employed respectively (Table 1).

Table1. Socio-demographic characteristics of pregnant women attended FGA Dessie model clinic, Northeast Ethiopia, 2019

Variable	Frequency (n=402)	Percentage (%)
**Marital status**		
Married	380	94.5
Others[a]	22	5.5
**Ethnicity**		
Amhara	361	89.8
Others[b]	41	10.2
**Income**		
≤5,000	203	50.5
5,001-10,000	163	40.5
>10,000	36	9.0
**Religion**		
Orthodox tewahido	216	53.7
Muslim	175	43.5

Protestant	11	2.7
**Residence**		
Urban	392	97.5
Rural	10	2.5
**Educational status**		
No formal education	17	4.2
Grade 1-8	65	16.2
Grade 9-12	124	30.8
College and above	196	48.8
**Occupation**		
Government employ	143	35.6
Private employ	48	11.9
Housewife	149	37.1
Others[c]	62	15.4

[a] single and divorced; [b] tigray and oromo; [c] farmer, merchant and daily laborer

Behavioral characteristics

One hundred thirty-one (32.6%) of pregnant women ever drink alcohol in their lifetime, 121(30.1%) of women drink alcohol in the last twelve months and 64 (15.9%) of women drink alcohol less than once a month in the last twelve month respectively. Seventy-one (17.7%) of pregnant women ever chew chat in their lifetime, 51(12.7%) of women chew chat in the last twelve months and 17 (4.2%) of women chew chat less than once a month in the last twelve month respectively. In the case of frequency of sexual intercourse, 71 (17.7%) and 219 (54.5%) of

pregnant women perform sexual intercourse three times a day and three times a week respectively (Table 2).

Table2. Behavioral characteristics of pregnant women attended FGA Dessie model clinic, Northeast Ethiopia, 2019

**Variables**	**Frequency (n=402)**	**Percentage (%)**
**Ever drink alcohol**		
Yes	131	32.6
No	271	67.4
**Alcohol use within 12 months**		
Yes	121	30.1
No	10	2.5
**Frequency of alcohol use**		
Daily	1	.2
One to four days per week	3	.7
One to three days per month	53	13.2
Less than once a month	64	15.9
**Ever chew chat**		
Yes	71	17.7
No	331	82.3
**Chew chat within 12 month**		
Yes	51	12.7

No	20	5.0
**Frequency of chewing**		
Monthly	5	1.2
Less than once a month	17	4.2
Rarely	29	7.2
**Frequency of sexual intercourse**		
Three times a day	71	17.7
Three times a week	219	54.5
Others[d]	112	27.9

[d], three times a month and three times a year

Contraceptive, obstetric and disease-related characteristics

Majority 172(42.8%) of pregnant women used Depo-Provera and 113 (28.1%) of women used implants as a contraceptive method before the current pregnancy. One hundred eighty-four (45.8%) of pregnant women used contraceptive methods for 12-24 months before the current pregnancy. Three hundred fifty-two (87.6%) of women give birth less than or equal to two times and 353(87.8%) of women had less than or equal to two children. Thirty (7.5%) of pregnant women had a history of abortion and 301(74.9%) of pregnant women had regular menstrual cycles before the current pregnancy. Four (1%) and 6(1.5%) of pregnant women had a history of Sexual Transmitted Infection (STI) and other medical illness (tuberculosis and diabetic Malthus) (Table 3).

Table3. Contraceptive, obstetric and disease-related characteristics of pregnant women attended FGA Dessie model clinic, Northeast Ethiopia, 2019

**Variables**	**Frequency (n=402)**	**Percentage (%)**
**Type of contraceptive used**		
OCP	91	22.6
Depo-Provera	172	42.8
Implant	113	28.1
IUCD	26	6.5
**Duration of contraceptive use**		
<12 month	107	26.6
12-24 month	184	45.8
25-36 month	63	15.7
>36 month	48	11.9
**Number of births**		
≤ 2	352	87.6
>2	50	12.4
**Number of children**		
≤ 2	353	87.8
>2	49	12.2
**History of abortion**		
Yes	30	7.5
No	372	92.5
**Menstrual cycle**		
Irregular	101	25.1

Regular	301	74.9
**History of STI**		
Yes	4	1.0
No	398	99.0
**History of medical illness**		
Yes	6	1.5
No	396	98.5

Proportion of fertility return

The proportion of delayed fertility return experienced by pregnant women after discontinuation of any modern contraceptive methods before the current pregnancy was 11.4% (95% CI; (8-14.4%)).

The proportion of delayed fertility return among pregnant women who used Depo-Provera, implant, IUCD, and OCP prior to the current pregnancy was 25% (95% CI; (19.2-31.4%)), 0.9% (95% CI; (0.001-2.7%)), 0% and 2.2% respectively.

The median time of fertility return among pregnant women after discontinuation of any modern contraceptive methods before the current pregnancy was 6 months with IQR of 8 months. The median time of fertility return among Depo-Provera implant, IUCD, and OCP users before the current pregnancy was 9 months, 4 months, 6 months, and 2 months with IQR of 7 months, 4 months, 8 months and 4 months respectively.

Factors associated with delayed fertility return

Both bi-variable and multivariable binary logistic regression analyses were done. The finding indicated that a unit increase in age resulted in delayed fertility return by 1.15 times (AOR= 1.15, (95% CI; (1.01, 1.30)). Pregnant women who used Depo-Provera before the current pregnancy

were 6.2 times more likely to experience fertility delay upon discontinuation as compared to women who used IUCD and OCP (AOR=6.22, (95% CI; 5.23, 13.3)) (Table 4).

Table 4. Factors associated with delayed fertility return after contraceptive discontinuation among pregnant women attended FGA Dessie Model Clinic, Northeast Ethiopia, 2019

**Variables**	**Fertility return (n=402)**		**COR (95% CI)**	**AOR (95% CI)**
	**Delayed**	**Not delayed**		
**Age**	46 (11.4%)	356(88.6%)	1.06 (0.98, 1.14)	**1.15 (1.01, 1.30)***
**Monthly Income**				
≤5,000	26 (56.5%)	177 (49.7%)	1	1
5,001-10,000	17 (37.0%)	146 (41.0%)	0.79 (0.41, 1.52)	1.00 (0.45, 2.23)
>10,000	3 (6.5%)	33 (9.3%)	0.62 (0.18, 2.16)	1.31 (0.28, 6.16)
**Educational status**				
No formal education	6 (13.0%)	11 (3.1%)	4.55(1.52, 13.6)*	2.52 (0.39, 16.17)
Grade 1-8	8 (17.4%)	57 (16.0%)	1.17 (0.49, 2.8)	1.47 (0.36, 5.99)
Grade 9-12	11 (23.9%)	113 (31.7%)	0.81 (0.38, 1.7)	0.52 (0.16, 1.72)
College and above	21 (45.7%)	175 (49.2%)	1	1
**Ever drink alcohol**				
Yes	15 (32.6%)	116 (32.6%)	1.01 (0.52, 1.9)	1.18 (0.51, 2.76)
No	31 (67.4%)	240 (67.4%)	1	1
Ever chew Khat				
Yes	11 (23.9%)	60 (16.9%)	1.55 (0.75, 3.2)	0.96 (0.27, 3.40)
No	35 (76.1%)	296 (83.1%)	1	1

**Duration of contraceptive use**				
<12 month	10 (21.7%)	97 (27.2%)	1	1
12-24 month	23 (50.0%)	161 (45.2%)	1.39 (0.63, 3.04)	0.95 (0.37, 2.42)
25-36 month	6 (13.0%)	57 (16.0%)	1.02 (0.35, 2.96)	1.26 (0.35, 4.52)
>36 month	7 (15.2%)	41 (11.5%)	1.66 (0.59, 4.65)	0.88 (0.23, 3.38)
**Number of live birth**				
≤ 2	39 (84.8%)	313 (87.9%)	0.77 (0.32, 1.82)	0.72 (0.06, 1.50)
>2	7 (15.2%)	43 (12.1%)	1	1
**Number of children**				
≤ 2	39 (84.8%)	314 (88.2%)	0.75 (0.31, 1.77)	0.12 (0.001, 1.76)
>2	7 (15.2%)	42 (11.8%)	1	1
**menstrual cycle**				
Irregular	19 (41.3%)	82 (23.0%)	2.4 (1.24,4.44)**	1.35 (0.59, 3.09)
Regular	27 (58.7%)	274 (77.0%)	1	1
**MUAC**	46 (11.4%)	356(88.6%)	0.92 (0.79, 1.07)	0.95 (0.78, 1.15)
**Occupation**				
Government employ	17 (37.0%)	126 (35.4%)	1	1
Private employ	4 (8.7%)	44 (12.4%)	0.67 (0.22, 2.11)	0.65 (0.16, 2.54)
House wife	14 (30.4%)	135 (37.9%)	0.77 (0.36, 1.62)	0.44 (0.12, 1.63)
Others[c]	11 (23.9%)	51 (14.3%)	1.60 (0.70, 3.65)	0.80 (0.21, 3.01)

**Frequency of sexual intercourse**				
Three times a day	10 (21.7%)	61 (17.1%)	1	1
Three times a week	24 (52.2%)	195 (54.8%)	0.751(0.34,1.68)	0.67 (0.19, 2.36)
Others[d]	12 (26.1%)	100 (28.1%)	0.732(0.34,1.80)	0.46 (0.11, 1.96)
**Type of contraceptive**				
Depo-provera	43 (93.5%)	129 (36.2%)	0.03(0.004, 0.2)*	**6.22 (5.2,13.3)*** **
Implants	1 (2.2%)	112 (31.5%)	0.05(0.01, 0.22)*	0.52 (0.04, 6.23)
Others [e]	2(4.3%)	115(32.3%)	1	1

COR, crude odds ratio; AOR, adjusted odds ratio; MUAC, middle-upper arm circumference; *significant at $P<0.05$; ** significant at $P<0.01$; *** significant at $P \leq 0.001$ in the bi-variable and multivariable logistic regression analysis; [c] merchant, farmer, and daily laborer; [d] three times a month and three times a year; [e] IUCD and OCP.

Discussion

The proportion of delayed fertility return experienced by pregnant women after discontinuation of any modern contraceptive methods before the current pregnancy was 11.4%. The proportion of delayed fertility return among Depo-Provera, implant, IUCD, and OCP users before the current pregnancy was (25%), (0.9%), 0% and (2.2%) respectively. Age and using Depo-Provera had a positive association with delayed fertility return.

The proportion of delayed fertility return (inability to conceive within twelve months) experienced by pregnant women after discontinuation of any modern contraceptive methods was 11% similar to a study done in England, which showed that 12 % of the participants experienced failure to conceive within one year after discontinuation of any contraceptive method [27]. The proportion

of delayed fertility return experienced by Depo-Provera users before the current pregnancy was 25% similar to another study which was 22.26% [19].

But, the proportion of delayed fertility return experienced by implant, IUCD, and OCP users was 0.9%, 0%, and 2.2%, respectively, which is low in contrast to a meta-analysis study which indicated that the proportion of delayed fertility return experienced by implant, IUCD, and OCP users was 25.3%, 15.25%, and 12.06% [19]. Similarly, the proportion of delayed fertility return experienced by IUCD users was also low as compared to a study done in the United States of America (USA) which was 19% [18]. This could be due to differences in the study setting and study population.

In this study, age has a significant association with delayed fertility return. A unit increase in age delay fertility return by 1.1 times. Different studies also revealed that as age increases fertility return will be delayed [27-29]. The reason behind this could be the number of eggs decreases as women get older due to a fixed number of eggs in the ovary. The other reason could be as age increases, women will be at higher risk of disorders that can affect fertility, such as uterine fibroids and endometriosis. Moreover, as age increases, the remaining eggs in older women are more likely to have abnormal chromosomes.

Using Depo-Provera had a positive association with delayed fertility return. A global handbook on family planning for health care providers demonstrated that Depo-Provera causes a delayed fertility return [15]. This could be due to Depo-Provera can stay in the body system longer than the other birth control methods, so that the clearance of progestin from the serum takes a long time and the meantime to ovulation become delayed. On the other hand, Depo-Provera causes excessive weight gain; this leads the women to stop ovulation and get irregular menstrual cycles.

Once women stop ovulation, they would not be able to conceive and therefore it delays fertility return.

This study has limitations. Some variables like history of alcohol use, chat chewing, duration of contraceptive use and duration of contraceptive discontinuation until the current pregnancy will be affected by recall bias. Being a facility-based study will also underestimate delayed fertility return as it is prone to miss defaulters and delayed visitors to their ANC appointment.

Conclusion

The proportion of delayed fertility return experienced by pregnant women after discontinuation of any modern contraceptive methods before the current pregnancy was consistent with other studies. But the proportion of delayed fertility return experienced by implant, IUCD, and OCP users before the current pregnancy was low. Age and using Depo-Provera had a positive association with delayed fertility return. Health care providers should provide detailed counseling for Depo-Provera users since it delays fertility return.

List of abbreviations

ANC, Antenatal Care; AOR, Adjusted odds ratio; CI, Confidence Interval; EDHS, Ethiopian Demographic and Health Survey; FGA, Family Guidance Association; GTP, Growth and Transformation Plan; IQR, Inter Quartile Range; IUCD, Uterine Contraceptive Device; OCP, Oral Contraceptive Pill; SDG, Sustainable Development Goal; STI, Sexual Transmitted Infection; USA, United States of America.

Authors' contribution

YD: involved in conception and design, data acquisition, data analysis, and interpretation**; BK**:

involved in Drafting the article and critically revising it for important intellectual content. **MA**: Drafting the article and critically revising it for important intellectual content; **MY**: Final approval of the version to be published; **BA**: Final approval of the version to be published; all the authors are Agreed to be accountable for all aspects of the work in ensuring that questions related to the accuracy or integrity of the work are appropriately investigated and resolved.

Acknowledgments

We would like to express our deepest gratitude to Wollo University for providing financial support to conduct this research. Finally, we would like to extend our appreciation for FGA Dessie Model Clinic Head and staff, study participants, data collectors, and supervisor for their cooperation during the data collection process.

References

1. Kaplan B, Nahum R, Yairi Y, Hirsch M, Pardo J, Yogev Y, et al. Use of various contraceptive methods and time of conception in a community-based population. European Journal of Obstetrics & Gynecology and Reproductive Biology. 2005; 123(1):72-76.

2. Spira A. Epidemiology of human reproduction. Human Reproduction. 1986; 1(2):111-115.

3. Jacobstein R. Long-acting and permanent contraception: an international development, service delivery perspective. Journal of midwifery & women's health. 2007; 52(4):361-367.

4. Kulier R, O'Brien P, Helmerhorst FM, Usher-Patel M, d'Arcangues C. Copper containing, framed intra-uterine devices for contraception. Cochrane database of systematic reviews. 2007(4).

5. Family Health International. Addressing Unmet Need for Family Planning in Africa; 2007.

6. Daling JR, Weiss NS, Metch BJ, Chow WH, Soderstrom RM, Moore DE, et al. Primary tubal infertility in relation to the use of an intrauterine device. New England journal of medicine. 1985;312(15):937-941.

7. Cramer DW, Schiff I, Schoenbaum SC, Gibson M, Belisle S, Albrecht B, et al. Tubal infertility and the intrauterine device. New England journal of medicine. 1985; 312(15):941-947.

8. Shearman R. Amenorrhea after treatment with oral contraceptives. Lancet (London, England). 1966;2(7473):1110.

9. Vessey M, Wright N, McPherson K, Wiggins P. Fertility after stopping different methods of contraception. Br Med J. 1978; 1(6108):265-267.

10. Linn S, Schoenbaum SC, Monson RR, Rosner B, Ryan KJ. Delay in conception for former'pill'users. Jama. 1982; 247(5):629-632.

11. Harlap S, Baras M. Conception-waits in fertile women after stopping oral contraceptives. International journal of fertility. 1984; 29(2):73-80.

12. Chasan-Taber L, Willett WC, Stampfer MJ, Spiegelman D, Rosner BA, Hunter DJ, et al. Oral contraceptives and ovulatory causes of delayed fertility. *American journal of epidemiology*. 1997; 146(3):258-265.

13. Fotherby K, Yong-En S, Howard G, Elder M, Muggeridge J. Return of ovulation and fertility in women using norethisterone oenanthate. Contraception. 1984; 29(5):447-455.

14. Pardthaisong T, Gray R, Mcdaniel E. Return of fertility after discontinuation of depot medroxyprogesterone acetate and intra-uterine devices in Northern Thailand. The lancet. 1980; 315(8167):509-512.

15. World Health Organization. Family planning: a global handbook for providers: 2011 update: evidence-based guidance developed through worldwide collaboration. 2011.

16. Vessey MP, Smith M, Yeates D. Return of fertility after discontinuation of oral contraceptives: influence of age and parity. Br J Fam Plann. 1986; 11(4):120-124.

17. Buck GM, Sever LE, Batt RE, Mendola P. Life-style factors and female infertility. Epidemiology. 1997:435-441.

18. Stoddard AM, Xu H, Madden T, Allsworth JE, Peipert JF. Fertility after intrauterine device removal: a pilot study. The European Journal of Contraception & Reproductive Health Care. 2015; 20(3):223-230.

19. Girum T, Wasie A. Return of fertility after discontinuation of contraception: a systematic review and meta-analysis. Contraception and reproductive medicine. 2018;3(1):9.

20. Wallach EE, Huggins GR, Cullins VE. Fertility after contraception or abortion. Fertility and sterility. 1990;54(4):559-573.

21. United Nation. Sutainable Developmental Goals. New York: United nation; 2016.

22. Federal Democratic Republic of Ethiopia. Growth and Transformation Plan draft. Ministry of Finance and Economic Development; 2014.

23. Family Care International: Save motherhood review. The Safe Motherhood Initiative 1987–2005. New York: Family Care International; 2007.

24. USAID. Update on family planning in Sub-Saharan Africa. Repositioning family planning: guidelines for advocacy action. Washington: Academy for Educational Development; 2009.

25. Smith R, Ashford L, Gribble J, Clifton D. Family planning saves lives. Washington DC: Population Reference Bureau. 2009;5.

26. Central Statistical Agency [Ethiopian and ICF International] Ethiopian Demographic Health Survey 2016. Addis Ababa, Ethiopia and Rockville, Maryland USA: Central Statistical Agency International;2016

27. Farrow A, Hull M, Northstone K, Taylor H, Ford W, Golding J. Prolonged use of oral contraception before a planned pregnancy is associated with a decreased risk of delayed conception. Human Reproduction. 2002;17(10):2754-2761.

28. Buckshee K, Chatterjee P, Dhall G, Hazra M, Kodkany B, Lalitha K, et al. Return of fertility following discontinuation of norplantR-II subdermal implants: ICMR task force on hormonal contraception. Contraception. 1995; 51(4):237-242.

29. Sivin I, Stern J, Diaz S, Pavéz M, Alvarez F, Brache V, et al. Rates and outcomes of planned pregnancy after use of Norplant capsules, Norplant II rods, or levonorgestrel-releasing or copper TCu 380Ag intrauterine contraceptive devices. American journal of obstetrics and gynecology. 1992; 166(4):1208-1213.

Supporting information

S1-Table: STROBE checklist

Click here to access/download
Supporting Information
S1 table.docx
