# Supplementary material for: Fertility return after hormonal contraceptive discontinuation and associated factors among women attended Family Guidance Association of Ethiopia Dessie model clinic, Northeast Ethiopia: A cross-sectional study
Source: PLoS One. 2023 Jul 11;18(7):e0287440. doi: 10.1371/journal.pone.0287440 (PMC10335688; doi:10.1371/journal.pone.0287440)
Supplement: Attachment — Submitted filename: Fertility return after contraceptive discontinuation Ethiopia_Review.docx [file pone.0287440.s005.docx]

The article needs to be edited for clarity. There are a lot of phrases that aren’t entirely clear and many details that are not included or are not elaborated upon sufficiently.

**Introduction**

The introduction section needs to more fully interrogate the existing evidence. The citations indicate several strong articles on the topic of return to fertility after contraceptive use; however, the introduction doesn’t summarize the science in a robust fashion.

The paragraph on page 3 (lines 54-56) is quite extreme. The association between impaired fertility and murder are not likely as strong as those between impaired fertility and mental health disorders. This paragraph should be revised to indicate that certain associations of impaired fertility are rare compared to others.

**Material and Methods**

Please provide background on FGA Dessie Model Clinic. It’s not clear what this is, who it serves, etc. Where is it? What context? What sorts of people go to this clinic- urban/peri-urban/rural? Stable population or migrating population? Did you collect data at first ANC visit or later ANC visit?

It would be useful to know more about the questionnaire- how many questions? Were the responses open-ended or categorized? If questionnaire derived from previous questionnaires from similar works of literature, it would be useful to cite them.

**Results**

It is unclear to me how decisions were made around categorizing Socio-demographic data, notably non-binary behavioral characteristics. I would like confirmation that nearly 18% of the study reports a frequency of sexual intercourse of three times per day- this seems questionable and could skew the results given the association between frequency of intercourse and fertility.

Please define further the variables. What is history of abortion- are you including spontaneous miscarriages in this definition? What is an irregular menstrual cycle? What would constitute history of medical illness- is it only TB and diabetes or inclusive of other illness? I’m curious about your cutoff for number of births, given that it would be interesting to better understand the distinction between those for whom this is their first pregnancy and those for whom its their second or greater- this is likely related to the sample, but bears noting.

**Discussion**

When citing previous studies, please note the authors, not only the location of the study.

Increasing age is associated with decreasing fertility. This is steeped in research. Please cite research on page 16 paragraph on lines 203-209. It currently reads in a speculative manner; however, in reality, these associations are real and documented.
