# Supplementary material for: Fertility return after hormonal contraceptive discontinuation and associated factors among women attended Family Guidance Association of Ethiopia Dessie model clinic, Northeast Ethiopia: A cross-sectional study
Source: PLoS One. 2023 Jul 11;18(7):e0287440. doi: 10.1371/journal.pone.0287440 (PMC10335688; doi:10.1371/journal.pone.0287440)
Supplement: Attachment — Submitted filename: Response to reviewers.docx [file pone.0287440.s007.docx]

Janet E Rosenbaum, Ph.D

Academic Editor, PLOS ONE

**RE: Submission ID** PONE-D-21-25440 R2 (Fertility return after contraceptive discontinuation and associated factors among women attended Family Guidance Association of Dessie model clinic, Northeast Ethiopia: a cross-sectional study)

**Dear Dr.** Janet E Rosenbaum**,**

Thank you very much for your email and the comments/suggestions of the reviewers and academic editor. We have looked at the comments and have revised our paper accordingly. We hope our paper improved as a result of incorporating the reviewers' and academic editor's comments and suggestions.

Please find for your kind consideration the following:

- A rebuttal letter that responds to each point raised by the academic editor and reviewer(s). You should upload this letter as a separate file labeled 'Response to Reviewers'.
- A marked-up copy of your manuscript that highlights changes made to the original version. You should upload this as a separate file labeled 'Revised Manuscript with Track Changes'.
- An unmarked version of your revised paper without tracked changes. You should upload this as a separate file labeled 'Manuscript'.

While hoping that these changes would meet with your favourable consideration, we are happy to hear if there are more comments and suggestions. Please do not hesitate to let us know if you have any questions.

Yours Sincerely,

Yitayish Damtie

School of Public Health, Wollo University

Dessie, Ethiopia

Tel:+251943517982

E-mail: [yitutile@gmail.com](mailto:yitutile@gmail.com)

**Point by point responses**

Reviewer #4: This work is interesting but data reported didn't give new information’s about fertility return after contraceptive discontinuation. This field has been largely studied and data reported in this work didn't add new information’s (1)

1 Girum T, Wasie A. Return of fertility after discontinuation of contraception: a systematic review and meta-analysis. Contraception Reprod Med. 2018 Jul 23;3:9. doi: 10.1186/s40834-018-0064-y. PMID: 30062044; PMCID: PMC6055351.

***Response: Thank you dear reviewer for your comment. Even if return of fertility after discontinuation of contraception has been largely studied and a systematic review and meta-analysis was done at the global level, the field has not been studied yet in Ethiopia. So, this study would have a paramount significance in providing important information for concerned individuals who are working to improve the reproductive health of Ethiopian women.***

Reviewer #5:

Title: Fertility return after contraceptive discontinuation and associated factors among women attended Family Guidance Association of Dessie model clinic, Northeast Ethiopia: a cross-sectional study

1. Abstract: Women who use hormonal contraception…... you have used hormonal contraception the reader will understand you have checked for only hormonal ones!!! Use it at the title also and be sure that when you are recording on the contraception within the text to be hormonal.

***Response: Thank you dear reviewer for your constructive comment. We have amended it.***

2. Abstract line 36: OCP includes combined oral contraception piles and progesterone only piles!!! please refer to it in a more definite way in all the text.

***Response: Thank you. As it is indicated in the abstract line 35 and throughout the manuscript, we used the abbreviation OCP to refer oral contraceptive pills which includes both combined oral contraception piles and progesterone only piles.***

3. Introduction, first line: Globally, around 15% of couples experience infertility!!! That is true; the proportion of delayed fertility return among currently pregnant women after a discontinuation contraceptive method was 11.4%in your research!! Do you think that there was any delay in getting pregnant? Almost 88% became pregnant within 1 year of attempting pregnancy.

***Response: Definitely! Although the figure is not high, 11.4% of currently pregnant women experienced a delayed fertility return after discontinuation of contraceptive methods before they became pregnant*.**

4. Introduction line 57-58: These include socio-demographic factors (age) [16]: socio-demographic factors does not means (age ) to put the word between bracket !! either add other factors with the age and this will need many references or delete it

***Response: Many thanks for your comment. We have amended it.***

5. Introduction (MMR, FGA) are two abbreviations needs to be proceeded by full description

***Response: Thank you for your comment. The full description of FGAE has already written in the abstract section so that we can use the abbreviated form in the subsequent sections. Regarding the abbreviation MMR, we removed it from the introduction due to other reviewer suggestion.***

6. Introduction line 67: One of the reasons for not using contraceptive methods might be due to fertility delay associated with contraceptive: At introduction you do not add your suggestions for a result!! You may shift it to the discussion section.

***Response: Thank you for your suggestion. We have revised it accordingly.***

7. Page 4 sample size: The sample size was determined by using single population proportion formula by considering the proportion of delayed fertility return as 50%
A. Using 50% reference is a very high percent!! Although there was no research done in this community , in these situations you may use the rate in nearby countries or even any published article on the same purpose

***Response: Thank you for your important comment. Many scholars recommended using 50% reference for sample size calculation in the case of unknown prevalence since it provides maximum sample size. It is not recommended to use the rate in nearby countries for sample size calculation due to the variation in the concern of government on health and health related issues and health care interventions across countries. In addition, the sample size calculated from studies conducted in nearby countries may not be larger than the sample size calculated using 50% reference for sample size since it provides maximum sample size. The* power of the study and *precision of the estimate* increases with an increase in sample size. *As long as it doesn’t affect the power and the precision of the study, it is better to use 50% reference in case of unknown prevalence.***

B. A cross sectional study means you will survey in a population about any one whom have used contraception and failed to get pregnant within one year so ideally does not need sample size estimation being a cross sectional study.

***Response: Sample size estimation is mandatory for any type of quantitative study including cross-sectional study. This is because if we survey a small number of women, we will not be able to detect an effect and may produce inconclusive result. On the other hand, if we survey large number of women, it may waste scares resources (time, money, manpower etc...). So, determination of a sample size which is an appropriate is crucial to avoid the aforementioned issues.***

C. Using single population proportion: which population due mean? Was it at antenatal care unites? Or gynecology outpatient clinic? Or may infertility unit?

***Response: Thank you for your comment. It is to mean antenatal care unites***.

8. Result: when I reached the Socio-demographic characteristics then understood that your participants were already pregnant now!!! And still didn’t get the setting of your research!!!!!

***Response: Really! Our study participants were women who are pregnant at the time of study. The aim of our study was to assess whether fertility was returned (whether women became pregnant) within 12 months of contraceptive discontinuation or not and to determine factors for those women who experience delayed in return of fertility after contraceptive discontinuation. Fertility return was defined if conception occurred within 12 months following discontinuation of any modern contraceptive methods. On the other hand, delayed fertility return was defined if conception occurred after 12 months following discontinuation of any modern contraceptive methods. So to achieve our objective, study participants should be pregnant women.***

9. Page 7, line 131: Ethiopian birr is a local currency which is not understandable for me and the readers!!! Please add to it how much it correspond to using a global currency like American dollar

***Response: Thank you for your important comment. We have tried to change it to American dollar.***

10. Page 8, line 141: chew khat!! I didn’t understand what it means!! Please clarify it in your methods section.

***Response: Thank you. To make it clear, Khat is a fresh green leave plant native to eastern Africa and the Arabian Peninsula. In 1980, WHO classified it as an illicit substance due to the potential for psychological dependence. Khat contains a psycho-active ingredient cathinone which is said to cause excitement, and*** [***euphoria***](https://en.wikipedia.org/wiki/Euphoria)***. Its regular consumption negatively impacts the human central nervous system, systemic blood pressure, psychological health and reproductive system causing reproductive toxicity and sexual dysfunction. We have tried to clarify it in the method section as well*.**

11. Page 9, line 144: Pregnant women perform sexual intercourse three times a day and three times a week respectively: what do you mean by this information? They are already pregnant and having intercourse many times does not give you any information about your objectives!!!

***Response: Yes, this study was conducted among women who are already pregnant. However, we used frequency of sexual intercourse as a factor to assess how often they performed sexual intercourse before they become pregnant since frequency of sexual intercourse is an important factor for the occurrence of pregnancy (fertility return). It is known that women who perform sexual intercourse three times a day, three times a week, three times a month and three times a year have no equal chance of becoming pregnant.***

12. Page 10, line 156: Four (1%) and 6(1.5%) of pregnant women had a history of Sexual Transmitted Infection (STI) and other medical illness (tuberculosis and diabetic Malthus). Although the delayed in getting pregnancy in your sample size was 11% (less than the rate of infertility all over the world (15%), still you have-not excluded whom already infertile from other causes like sexually transmitted disease and uncontrolled diabetes mellitus!! you have to clarify this point or to remove the 4 ladies having other causes for infertility not delay in getting pregnancy after withdrawal of contraception.

***Response: Thank you for your comment. Our study participants were women who are pregnant at the time of study. Infertility could not be an issue for our study participants since they are already pregnant. Studies suggested that having STI and other medical illness like Tuberculosis and diabetic Malthus can delay pregnancy or may causes infertility if they are left untreated. Due to these, we want to test their statistical significance association with delay in getting pregnancy.***
